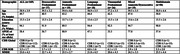# Heterogeneous clinical phenotypes of sporadic Early‐onset Alzheimer’s disease: A data‐driven approach

**DOI:** 10.1002/alz.091639

**Published:** 2025-01-03

**Authors:** Deepti Putcha, Yuta Katsumi, Alexandra Touroutoglou, Jeffrey L. Dage, Ani Eloyan, Kelly N. Nudelman, Maria C. Carrillo, Gil D. Rabinovici, Liana G. Apostolova, Bradford C. Dickerson, Dustin B. Hammers, LEADS Consortium

**Affiliations:** ^1^ Frontotemporal Disorders Unit, Department of Neurology, Massachusetts General Hospital and Harvard Medical School, Boston, MA USA; ^2^ Department of Neurology, Indiana University School of Medicine, Indianapolis, IN USA; ^3^ Department of Medical and Molecular Genetics, Indiana University School of Medicine, Indianapolis, IN USA; ^4^ Department of Biostatistics, Brown University, Providence, RI USA; ^5^ Alzheimer’s Association, Chicago, IL USA; ^6^ Department of Neurology, Memory and Aging Center, University of California San Francisco, San Francisco, CA USA; ^7^ Department of Radiology and Imaging Sciences, Indiana University School of Medicine, Indianapolis, IN USA; ^8^ Indiana University School of Medicine, Indianapolis, IN USA

## Abstract

**Background:**

Early‐onset Alzheimer’s disease (EOAD) manifests prior to the age of 65. Clinical presentation of EOAD is distinct from that of late‐onset Alzheimer’s disease, and is characterized as having a more aggressive disease course with greater heterogeneity. Recent publications from the Longitudinal Early‐Onset Alzheimer’s Disease Study (LEADS) described their sample as predominantly amnestic, though this phenotypic description was based solely on clinical judgment. To better understand the range of EOAD presentation, we applied a neuropsychological data‐driven method to phenotypic subtyping within the LEADS cohort.

**Method:**

Data from 169 amyloid‐positive EOAD participants with composite data in all cognitive domains (Episodic Memory, Executive Functioning, Speed/Attention, Language, and Visuospatial) were analyzed. Our approach consisted of comparing the relative levels of baseline impairment in each cognitive domain. Education‐corrected normative comparisons were made using a sample of 98 aged‐matched cognitively normal participants. A cut‐off of 1 *SD* below all other composite domain scores was applied to indicate a phenotype of “predominant” impairment in a given cognitive domain. Individuals were otherwise considered to have a phenotype best characterized by multidomain impairment.

**Result:**

We identified 6 phenotypic subtypes of EOAD (Table 1): Dysexecutive‐predominant (22% of sample), Amnestic‐predominant (11%), Language‐predominant (11%), Visuospatial‐predominant (15%), Mixed Amnestic/Dysexecutive‐predominant (11%), and Multidomain (30%). These subtypes did not differ on age, age‐at‐symptom‐onset, sex, or overall clinical severity (*p*>0.05). Groups differed on global cognitive functioning (MMSE) such that the Amnestic‐predominant group performed better than other domain‐predominant subtypes of EOAD (*p*>0.05). In contrast to the heterogeneity observed from our data‐driven approach, diagnostic classifications for this same sample based solely on clinical judgment indicated that 82% of individuals were amnestic‐predominant, 9% were non‐amnestic, 4% were visuospatial‐predominant, and 5% were language‐predominant.

**Conclusion:**

Applying a neuropsychological data‐driven method of phenotyping EOAD individuals uncovered a more detailed understanding of the diversity of presenting heterogeneity in this atypical AD group compared to clinical judgment alone. These results suggest that clinicians and patients may over‐prioritize memory dysfunction during subjective reporting at the expense of non‐memory symptoms, which has important implications for diagnostic accuracy and treatment considerations. We plan to investigate the patterns of cortical atrophy and network dysfunction subserving this heterogeneity.